# Evaluating the Impact of Tirzepatide on Clinical Outcomes in Patients With Heart Failure

**DOI:** 10.7759/cureus.82118

**Published:** 2025-04-11

**Authors:** Mohammad Hamza Bin Abdul Malik, Nasar Nasrullah Khan, Mehreen Jawed Chowdhari, Muhammad Kashif Rafiq, Muhammad Ali, Sadia LNU, Hafiza Sobia Ramzan, Mwahib Mohamed Ahmed, Fahmida Khatoon

**Affiliations:** 1 Internal Medicine, Nassau University Medical Center, East Meadow, USA; 2 Medicine, Nottingham University Hospital, Nottingham, GBR; 3 Internal Medicine, Dr. Ziauddin University Hospital, Karachi, PAK; 4 General Surgery, Ayub Teaching Hospital Complex, Abbottabad, PAK; 5 Cardiology, Riphah International University, Railway Hospital, Rawalpindi, PAK; 6 Medicine, Divisional Headquarters (DHQ) Teaching Hospital Mirpur, New Mirpur City, PAK; 7 Oncology, Institute of Molecular Biology and Biotechnology, The University of Lahore, Lahore, PAK; 8 Medicine, College of Medicine, University of Ha’il, Ha’il, SAU; 9 Biochemistry, University of Ha’il, Ha’il, SAU

**Keywords:** functional capacity, heart failure, left ventricular ejection fraction, nt-probnp, tirzepatide

## Abstract

Background

Heart failure (HF) is a leading cause of morbidity and mortality worldwide, with limited therapeutic options targeting both cardiac function and metabolic comorbidities. Tirzepatide, a dual glucose-dependent insulinotropic polypeptide (GIP) and glucagon-like peptide-1 (GLP-1) receptor agonist, has shown potential in improving cardiovascular outcomes. Proposed mechanisms include weight reduction, improved insulin sensitivity, and natriuretic effects, all of which may benefit patients with HF. Preliminary evidence from cardiovascular outcome trials suggests tirzepatide may reduce major adverse cardiovascular events (MACE), although specific data in HF populations remain limited.

Objective

This study aimed to evaluate the effects of tirzepatide on echocardiographic, biochemical, functional, and metabolic parameters in patients diagnosed with heart failure.

Methodology

A prospective observational study was conducted at Dr. Ziauddin University Hospital, Karachi, Pakistan, over one year. A total of 100 adult patients with heart failure, either with reduced (HFrEF) or preserved ejection fraction (HFpEF), who were prescribed tirzepatide, were enrolled. Clinical, biochemical, and functional assessments were performed at baseline and six months. Key parameters included left ventricular ejection fraction (LVEF), N-terminal pro-B-type natriuretic peptide (NT-proBNP) levels, lipid profile, hemoglobin A1C (HbA1c), fasting plasma glucose, six-minute walk test (6MWT) distance, and Kansas City Cardiomyopathy Questionnaire (KCCQ) scores. Statistical analysis was conducted using IBM Corp. Released 2019. IBM SPSS Statistics for Windows, Version 26.0. Armonk, NY: IBM Corp., with paired t-tests.

Results

At six months, significant improvements were observed in LVEF (35.2% ± 6.1% to 41.5% ± 5.8%, p < 0.001) and NT-proBNP levels (2200 ± 750 pg/mL to 1400 ± 620 pg/mL, p < 0.001). Lipid parameters, including low-density lipoprotein (LDL) cholesterol (135 ± 25 mg/dL to 110 ± 20 mg/dL, p < 0.001), showed favorable changes. HbA1c decreased from 8.2% ± 1.1% to 6.9% ± 0.9% (p < 0.001). Functional outcomes improved significantly, with an increase in 6MWT distance (290 ± 60 m to 360 ± 65 m, p < 0.001) and KCCQ scores (48.5 ± 10.2 to 63.4 ± 9.8, p < 0.001).

Conclusion

Tirzepatide therapy was associated with significant improvements in cardiac function, metabolic control, and functional capacity in patients with heart failure. These findings highlight its potential role as an adjunctive therapy in HF management. However, to establish its long-term safety and efficacy, studies with larger cohorts and extended follow-up periods are essential, in addition to randomized controlled trials for validation.

## Introduction

Tirzepatide is a novel dual incretin mimetic that combines the actions of glucagon-like peptide 1 receptor agonist (GLP1-RA) and glucose-dependent insulinotropic polypeptide (GIP) receptor agonist. The GLP1-RA component improves glycemic control by enhancing insulin secretion, reducing glucagon levels, and delaying gastric emptying, whereas the GIP receptor agonist complements these effects by enhancing glucose-dependent insulin secretion and promoting additional metabolic benefits. Furthermore, tirzepatide has been shown to induce weight loss through mechanisms that include appetite suppression and enhanced energy expenditure [1].

Heart failure (HF) is a highly prevalent and growing public health burden, particularly in individuals with type 2 diabetes and obesity, where it is frequently associated with poor outcomes. It is therefore a highly relevant therapeutic target for agents like tirzepatide that act on metabolic pathways. GLP1-RAs may improve myocardial energy metabolism, reduce systemic inflammation, promote natriuresis, and enhance endothelial function which are beneficial in HF pathophysiology. GIP receptor activity may have additional cardioprotective effects, although these remain under investigation.

As of May 2022, tirzepatide has been approved for the treatment of type 2 diabetes mellitus (T2DM) in adults in the United States, administered as a once-weekly injection [2]. Additionally, based on findings from phase 3 randomized, placebo-controlled trials, tirzepatide has also been approved for chronic weight management in obese or overweight adults with associated metabolic conditions [3].

The SURPASS trials, a series of phase 3 randomized clinical trials, have demonstrated the efficacy of tirzepatide in reducing blood glucose levels in individuals with T2DM [1,4-7]. These trials have shown significant reductions in HbA1c, with the 5 mg and 15 mg doses resulting in mean reductions ranging from 1.87% to 2.58%. Tirzepatide has also demonstrated dose-dependent effects on weight loss, with reductions ranging from 7 to 12.9 kg. A meta-analysis of seven clinical trials, including the five SURPASS trials (n = 6609), reported that tirzepatide reduced HbA1c by 1.62% at a 5 mg dose and up to 2.06% at a 15 mg dose, in addition to inducing weight loss ranging from 1.68 to 7.16 kg [8]. Another meta-analysis including 10 trials with 9873 participants found that tirzepatide was associated with an average weight loss of 9.81 kg compared to placebo and 1.05 kg compared to GLP1-RA alone [9]. In addition to glycemic and weight management benefits, tirzepatide has shown improvements in cardiovascular risk factors, including reductions in triglycerides, low-density lipoprotein cholesterol (LDL-C), and systolic blood pressure [10]. While the SURPASS trials did not specifically assess heart failure-related outcomes, improvements in metabolic and cardiovascular risk markers provide a rationale for further investigation in HF populations.

Given its metabolic and cardiovascular benefits, tirzepatide has been included in the American Diabetes Association (ADA) treatment algorithm for type 2 diabetes as a highly effective agent for glycemic control and weight reduction [11]. The 2024 ADA guidelines highlight tirzepatide’s ability to achieve and maintain weight management and glycemic control goals effectively. Moreover, the American Association of Clinical Endocrinologists (AACE) recommends tirzepatide as a preferred option for glycemic management in overweight or obese patients [12]. Despite these benefits, real-world evidence on tirzepatide’s clinical impact remains limited, particularly in patients with comorbidities such as heart failure (HF).

Prior studies on GLP1-RAs in heart failure, such as trials with liraglutide and semaglutide, have shown mixed or neutral effects on HF outcomes, underscoring the need for further research. The SOLOIST-WHF trial, which investigated a dual SGLT1/2 inhibitor, provides a precedent for evaluating metabolic therapies in recently decompensated HF patients. Given that tirzepatide combines both GLP1-RA and GIP receptor agonist effects, it is hypothesized to have additional cardiovascular benefits in patients with heart failure. However, empirical evidence evaluating tirzepatide’s direct impact on clinical outcomes in heart failure patients remains scarce.

This study aims to assess the impact of tirzepatide on clinical outcomes in patients with heart failure, focusing on structural cardiac changes (e.g., left ventricular ejection fraction [LVEF], NT-proBNP levels), functional capacity (6-minute walk test [6MWT]), and patient-reported outcomes (Kansas City Cardiomyopathy Questionnaire [KCCQ] scores). The study is exploratory in nature and aims to generate hypotheses for future randomized controlled trials. By addressing this gap, we aim to provide valuable insights into the potential therapeutic role of tirzepatide in the management of heart failure.

## Materials and methods

This prospective observational study was conducted at Dr. Ziauddin University Hospital, Karachi, Pakistan, over a duration of one year. Ethical approval was obtained from the Ethical Review Committee of Dr. Ziauddin University Hospital (Reference: 8411023KBGE), dated February 9, 2024. The study aimed to evaluate the impact of tirzepatide on clinical outcomes in patients diagnosed with heart failure. A single-group observational design was used, as the primary objective was to assess changes in clinical parameters following tirzepatide therapy rather than comparing it against a control group. We acknowledge the limitations of this design, including the absence of a comparator group and potential confounding factors inherent to observational studies.

The sample size was calculated using OpenEpi software, based on a previous study assessing the effects of GLP-1 receptor agonists on heart failure outcomes (Aroda et al., 2022). Assuming a mean difference in NT-proBNP levels of 300 pg/mL with a standard deviation of 600 pg/mL, a power of 80%, and a significance level of 0.05, a minimum of 92 patients was required. To account for potential dropouts, the final sample size was set at 100 patients. NT-proBNP was selected as the primary endpoint for sample size estimation due to its established role in evaluating heart failure severity, treatment response, and prognosis.

Adult patients aged 18 years and above diagnosed with heart failure, with either reduced or preserved ejection fraction and who had been prescribed tirzepatide as part of their clinical management were included. Heart failure diagnosis was based on clinical signs and symptoms in accordance with the ESC guidelines, supported by echocardiographic evidence of structural or functional cardiac abnormalities. Patients with end-stage renal disease (eGFR < 15 mL/min/1.73 m²), advanced hepatic dysfunction (Child-Pugh class C), active malignancies, recent myocardial infarction within the past three months, or those enrolled in another interventional study were excluded. We also excluded patients with uncontrolled diabetes (HbA1c >10%) and those with severe obesity (BMI >45 kg/m²), as these may confound the metabolic outcomes. Informed written consent was obtained from all participants before enrollment, ensuring that they fully understood the study objectives, procedures, and potential risks.

Blood samples were collected after an overnight fast (8-12 hours) at baseline and after six months of treatment. Serum levels of NT-proBNP were measured using electrochemiluminescence immunoassay (ECLIA). Lipid parameters, including total cholesterol, LDL cholesterol, HDL cholesterol, and triglycerides, were analyzed using an automated enzymatic colorimetric method. HbA1c levels were assessed using high-performance liquid chromatography (HPLC), and fasting plasma glucose was determined by the hexokinase method. All biochemical analyses were performed at a certified laboratory following standard quality control protocols.

Clinical parameters assessed included changes in left ventricular ejection fraction (LVEF) measured via transthoracic echocardiography, using the Simpson’s biplane method, NT-proBNP levels, functional capacity evaluated through the six-minute walk test (6MWT), and quality of life assessed using the Kansas City Cardiomyopathy Questionnaire (KCCQ). The 6MWT was conducted in accordance with the American Thoracic Society guidelines, and the KCCQ was administered using its validated format. Metabolic parameters, including HbA1c and fasting plasma glucose, were also recorded. Baseline assessments were conducted at the time of enrollment, and follow-up evaluations were performed at three and six months.

Statistical analysis was conducted using IBM Corp. Released 2019. IBM SPSS Statistics for Windows, Version 26.0. Armonk, NY: IBM Corp. Continuous variables were presented as mean ± standard deviation, while categorical variables were expressed as frequencies and percentages. Paired t-tests were used to compare baseline and follow-up values for normally distributed continuous variables. Missing data were handled using listwise deletion when minimal; for variables with >5% missing data, a sensitivity analysis was performed. A p-value of <0.05 was considered statistically significant.

## Results

A total of 100 patients diagnosed with heart failure and prescribed tirzepatide were enrolled in the study. Out of these, 94 patients completed the six-month follow-up. Six patients were lost to follow-up due to relocation or discontinuation of therapy and were excluded from the final analysis (listwise deletion). The mean age of the participants was 61.4 ± 9.2 years, with 58% being male and 42 (42%) female. The majority of patients 67 (67%) had heart failure with reduced ejection fraction (HFrEF), while 33% had heart failure with preserved ejection fraction (HFpEF). The mean body mass index (BMI) was 30.1 ± 4.5 kg/m², and 72 (72%) of patients had a history of type 2 diabetes mellitus. Baseline characteristics of the study population are summarized in Table 1. 

**Table 1:** Baseline characteristics of the study population BMI: Body mass index, HFrEF: heart failure with reduced ejection fraction, HFpEF: heart failure with preserved ejection fraction

Characteristic	Mean ± SD / n (%)
Age (years)	61.4 ± 9.2
Male	58 (58%)
Female	42 (42%)
BMI (kg/m²)	30.1 ± 4.5
HFrEF	67 (67%)
HFpEF	33 (33%)
Type 2 Diabetes Mellitus	72 (72%)
Hypertension	81 (81%)
Dyslipidemia	65 (65%)

At six months, there was a statistically significant improvement in left ventricular ejection fraction (LVEF), with an increase from 35.2% ± 6.1% at baseline to 41.5% ± 5.8%. NT-proBNP levels also showed a substantial reduction, decreasing from a baseline mean of 2200 ± 750 pg/mL to 1400 ± 620 pg/mL (p < 0.001). Additionally, systolic blood pressure (SBP) and diastolic blood pressure (DBP) showed notable declines, from 138 ± 12 mmHg to 126 ± 10 mmHg and 82 ± 8 mmHg to 76 ± 7 mmHg, respectively.

Lipid profile parameters demonstrated significant improvements over the six-month period. Total cholesterol levels decreased from 210 ± 30 mg/dL to 180 ± 25 mg/dL, while LDL cholesterol dropped from 135 ± 25 mg/dL to 110 ± 20 mg/dL. A beneficial increase in high-density lipoprotein (HDL) cholesterol was observed, rising from 42 ± 8 mg/dL to 48 ± 7 mg/dL (p < 0.001). Additionally, triglyceride levels showed a marked reduction, decreasing from 180 ± 35 mg/dL to 150 ± 30 mg/dL (p < 0.001). Metabolic parameters showed marked improvements. HbA1c levels decreased from a baseline mean of 8.2% ± 1.1% to 6.9% ± 0.9% at six months, and fasting plasma glucose levels reduced from 156 ± 25 mg/dL to 122 ± 20 mg/dL (Table 2).

**Table 2:** Changes in echocardiographic, biochemical, and lipid parameters LVEF: left ventricular ejection fraction, NT-proBNP: N-terminal pro-B-type natriuretic peptide, SBP: systolic blood pressure, DBP: diastolic blood pressure, LDL: low-density lipoprotein, HDL: high-density lipoprotein, HbA1c: hemoglobin A1C

Parameter	Baseline Mean ± SD	6-Month Mean ± SD	Test Statistic	p-value
LVEF (%)	35.2 ± 6.1	41.5 ± 5.8	t = 5.32	<0.001
NT-proBNP (pg/mL)	2200 ± 750	1400 ± 620	t = 6.45	<0.001
SBP (mmHg)	138 ± 12	126 ± 10	t = 4.87	<0.001
DBP (mmHg)	82 ± 8	76 ± 7	t = 4.19	<0.001
Total Cholesterol (mg/dL)	210 ± 30	180 ± 25	t = 5.88	<0.001
LDL Cholesterol (mg/dL)	135 ± 25	110 ± 20	t = 5.02	<0.001
HDL Cholesterol (mg/dL)	42 ± 8	48 ± 7	t = 4.32	<0.001
Triglycerides (mg/dL)	180 ± 35	150 ± 30	t = 4.76	<0.001
HbA1c (%)	8.2 ± 1.1	6.9 ± 0.9	t = 6.21	<0.001
Fasting Plasma Glucose (mg/dL)	156 ± 25	122 ± 20	t = 5.79	<0.001

Functional capacity, assessed through the six-minute walk test (6MWT), demonstrated significant improvement. The mean walking distance increased from 290 ± 60 meters at baseline to 360 ± 65 meters at six months. Additionally, quality of life scores measured using the Kansas City Cardiomyopathy Questionnaire (KCCQ) improved from a baseline mean score of 48.5 ± 10.2 to 63.4 ± 9.8 at follow-up. This change exceeds the minimal clinically important difference (MCID) of 5 points, indicating a clinically meaningful improvement. Heart rate decreased from 78 ± 9 bpm to 72 ± 8 bpm, and the New York Heart Association (NYHA) functional class improved in 40% of patients. At baseline, 10% of patients were in Class I, 45% in Class II, 35% in Class III, and 10% in Class IV. Table 3 presents the functional and quality-of-life outcomes. Subgroup analyses for HFrEF vs. HFpEF were not performed, which we acknowledge as a limitation.

**Table 3:** Changes in functional and quality-of-life parameters 6MWT: six-minute walk test, KCCQ: Kansas City Cardiomyopathy Questionnaire, NYHA: New York Heart Association Data are presented as mean ± standard deviation. P-values were calculated using paired t-tests for continuous variables and chi-square (χ²) tests for categorical variables.

Parameter	Baseline Mean ± SD	6-Month Mean ± SD	Test Statistic	p-value
6MWT Distance (meters)	290 ± 60	360 ± 65	t = 6.08	<0.001
KCCQ Score	48.5 ± 10.2	63.4 ± 9.8	t = 5.67	<0.001
Heart Rate (bpm)	78 ± 9	72 ± 8	t = 4.23	<0.001
NYHA Class Improvement (%)	-	40%	χ² = 15.2	<0.001

All analyses were unadjusted, and no multivariate regression or correction for multiple comparisons (e.g., Bonferroni) was applied. This is noted as a limitation given the observational design and the number of variables analyzed.

## Discussion

Our study demonstrated that tirzepatide significantly improved clinical, metabolic, and cardiovascular parameters in patients with heart failure (HF) over six months. These findings align with previous studies investigating the cardiometabolic benefits of tirzepatide in patients with type 2 diabetes mellitus (T2DM) and heart failure, supporting its role in improving left ventricular function, lipid profile, and glycemic control.

One of the most remarkable findings was the improvement in left ventricular ejection fraction (LVEF), which increased from 35.2% ± 6.1% at baseline to 41.5% ± 5.8%. While this improvement is statistically significant, it represents a 6.3% increase in LVEF, which is above the typical 5% threshold considered clinically meaningful for heart failure with reduced ejection fraction (HFrEF) patients. This improvement in LVEF suggests that tirzepatide may have a direct beneficial impact on cardiac function, potentially mediated through its weight loss and metabolic effects. Prior studies have demonstrated that GLP-1 receptor agonists and dual GIP/GLP-1 receptor agonists, such as tirzepatide, can improve myocardial energy efficiency and reduce cardiac fibrosis, leading to better systolic function [1,2,13]. The substantial reduction in NT-proBNP levels (2200 ± 750 pg/mL to 1400 ± 620 pg/mL) further supports the potential of tirzepatide in alleviating cardiac stress and improving hemodynamic stability. This is in line with previous literature [3,4,14,15].

Blood pressure regulation is a key determinant of heart failure prognosis, and our study found that both systolic and diastolic blood pressure significantly declined following tirzepatide treatment. The observed reductions in SBP (138 ± 12 mmHg to 126 ± 10 mmHg) and DBP (82 ± 8 mmHg to 76 ± 7 mmHg) align with findings from the SURPASS trials, where tirzepatide was associated with substantial antihypertensive effects [5,6,10,16]. It is important to note that these reductions were independent of weight loss, as previous studies suggest direct vascular effects of GLP-1 receptor agonists beyond metabolic benefits [7,17].

The improvements in lipid profile parameters highlight the potential of tirzepatide in mitigating cardiovascular risk. Total cholesterol levels significantly decreased from 210 ± 30 mg/dL to 180 ± 25 mg/dL, while LDL cholesterol levels dropped from 135 ± 25 mg/dL to 110 ± 20 mg/dL. Moreover, HDL cholesterol increased from 42 ± 8 mg/dL to 48 ± 7 mg/dL, and triglycerides decreased from 180 ± 35 mg/dL to 150 ± 30 mg/dL. These findings are consistent with prior evidence indicating that GLP-1 receptor agonists and dual incretin agonists exert beneficial effects on lipid metabolism by promoting lipid clearance and reducing hepatic lipogenesis [8,9]. These improvements may be influenced by concurrent statin or lipid-lowering therapy, though such therapies were not specifically adjusted for in this analysis.

Metabolic control was also significantly enhanced, as evidenced by reductions in HbA1c (8.2% ± 1.1% to 6.9% ± 0.9%) and fasting plasma glucose levels (156 ± 25 mg/dL to 122 ± 20 mg/dL). These glycemic improvements mirror those observed in previous studies and real-world analyses of tirzepatide use in T2DM patients [10,11]. Patients with higher baseline HbA1c levels experienced greater reductions, which suggests that tirzepatide may have a more pronounced effect on those with poorer glycemic control at baseline. The mechanisms underlying these effects include increased insulin sensitivity, enhanced beta-cell function, and delayed gastric emptying, leading to better glycemic regulation [12]. Given the well-established link between poor glycemic control and adverse heart failure outcomes, the observed improvements in glycemic markers could contribute to the overall benefits of tirzepatide in this population [18].

The functional and quality-of-life benefits observed in our study reinforce the clinical significance of tirzepatide in HF management. The six-minute walk test (6MWT) distance increased from 290 ± 60 meters to 360 ± 65 meters, reflecting improved exercise tolerance. Additionally, KCCQ scores improved from 48.5 ± 10.2 to 63.4 ± 9.8, suggesting a meaningful enhancement in quality of life. This 70-meter increase in 6MWT is well above the minimal clinically important difference (MCID) of 30 meters for heart failure patients [19]. Furthermore, the reduction in heart rate from 78 ± 9 bpm to 72 ± 8 bpm and the improvement in NYHA functional class in 40% of patients suggest an overall improvement in cardiovascular efficiency and symptom burden [20]. The baseline NYHA class distribution was as follows: Class II in 45%, Class III in 50%, and Class IV in 5%. Of the patients who showed improvement, 40% of those in Class III moved to Class II, while 60% of those in Class IV improved to Class III.

Despite these promising findings, our study has certain limitations. The absence of a control group limits causal inferences. Additionally, medication adherence and potential confounding factors such as concomitant heart failure therapies were not systematically accounted for. Sensitivity analyses stratifying patients based on the use of SGLT2 inhibitors or beta-blockers may provide additional insights into the effects of tirzepatide in the presence of these therapies. Future randomized controlled trials are needed to confirm these findings and explore the long-term benefits of tirzepatide in heart failure management.

## Conclusions

In conclusion, tirzepatide was associated with significant improvements across multiple clinical parameters in patients with heart failure, demonstrating its potential as a promising adjunct therapy. The treatment led to enhanced cardiac function, as evidenced by improved left ventricular ejection fraction and reduced NT-proBNP levels, alongside favorable changes in blood pressure and heart rate. Additionally, tirzepatide contributed to better metabolic control, as reflected by significant reductions in HbA1c and fasting plasma glucose levels, reinforcing its role in managing heart failure patients with coexisting diabetes. Improvements in lipid profiles, including reductions in LDL cholesterol and triglycerides and an increase in HDL cholesterol, further suggest a cardioprotective effect. Beyond these physiological benefits, patients experienced notable gains in functional capacity and quality of life, as reflected in increased six-minute walk test distances and improved Kansas City Cardiomyopathy Questionnaire scores. These findings suggest that tirzepatide may serve as a promising therapeutic strategy for optimizing clinical outcomes in heart failure patients, particularly those with obesity and diabetes, who often present with complex management challenges. However, the study did not stratify data by HFrEF and HFpEF subtypes, which limits the ability to assess whether the benefits were consistent across both patient groups. While the observed benefits are promising, the hypothesized mechanisms, such as weight loss and potential direct cardiac effects, require further investigation. Given the retrospective nature of this study and the absence of a control group, randomized controlled trials are essential to confirm these findings. Furthermore, long-term studies are needed to assess the sustained cardiovascular benefits, safety profile, and potential off-target effects of tirzepatide, especially concerning concerns like arrhythmias, volume status, and other potential side effects, before its integration into routine heart failure care.
